# Neuromodulation and mindfulness as therapeutic treatment in detoxified patients with alcohol use disorder

**DOI:** 10.1186/s12888-024-06085-4

**Published:** 2024-09-27

**Authors:** Annika Rosenthal, D. Haslacher, M. Garbusow, L. Pangratz, B. Apfel, S. Soekadar, N. Romanczuk-Seiferth, A. Beck

**Affiliations:** 1 Institute for Mental Health and Behavioral Medicine, Department of Psychology, HMU Health and Medical University Potsdam, 14471 Potsdam, Germany; 2 Department of Psychiatry and Neurosciences, Charité - Universitätsmedizin Berlin, Corporate Member of Freie Universität Berlin, Humboldt-Universität zu Berlin, Campus Charité Mitte, 10117 Berlin, Germany; 3 Department of Psychology, MSB Medical School Berlin, 14197 Berlin, Germany

**Keywords:** Mindfulness-based relapse prevention, Alcohol use disorder, Cue reactivity, Cognitive control, Interception, Frontal midline theta, Neuromodulation, Vagus nerve stimulation, Heart rate variability, Closed-loop amplitude-modulated transcranial alternating current stimulation, Electroencephalography

## Abstract

**Background:**

Alcohol use disorder (AUD) poses a significant global health challenge. Traditional management strategies often face high relapse rates, leading to a need for innovative approaches. Mindfulness-based relapse prevention (MBRP) has emerged as a promising intervention to enhance cognitive control, reduce cue-related craving and improve interoceptive processing. Neuroimaging studies suggest that mindfulness training can modulate brain networks associated with these factors, potentially improving treatment outcomes for AUD. Neuroimaging studies suggest that mindfulness training can modulate brain networks linked to these brain functions, potentially improving treatment outcomes for AUD. However, it is unclear how MBRP links to neurophysiological measures such as frontal midline theta oscillations (FMΘ) and whether the beneficial effects of MBRP can be increased by enhancing FMΘ. Here, we will use two different forms of neuromodulation to target and enhance these oscillations, and evaluate their impact on the effectiveness of MBRP.

**Methods:**

This study will employ a four-arm randomized controlled trial to evaluate the synergistic effects of MBRP augmented with transcutaneous vagus nerve stimulation (tVNS) or closed-loop amplitude-modulated transcranial alternating current stimulation (CLAM-tACS) on cognitive control, cue reactivity and interoceptive processing in AUD patients. Participants will undergo six weekly group MBRP sessions and daily individual mindfulness practices. Assessments will include an inhibition task, cue-induced craving task, and heartbeat discrimination task, alongside heart rate variability and 32-channel EEG recordings. Participants will be assessed pre and post treatment, with a three-month follow-up to evaluate long-term effects on abstinence and alcohol consumption.

**Discussion:**

This study will not only elucidate the causal link between FMΘ and efficacy of MBRP, but contribute to a better understanding of how combined psychological and neuromodulation interventions can improve treatment outcomes for AUD, potentially leading to more effective therapeutic strategies. This study also seeks to explore individual differences in response to treatment, which could inform future approaches to AUD management.

**Trial registration:**

This study received approval by the Charité—Universitätsmedizin Berlin Institutional Review Board (EA1/030/23, 10.11.2023). It was registered on ClinicalTrials.gov (NCT06308484).

## Background

Alcohol use disorder (AUD) is a significant global health concern, contributing to about 5% of the global disease burden, with an estimated 3.8% of global deaths attributable to it [1, 2]. Despite its prevalence, with 12-month rates ranging from 5 to 14% [3], AUD management faces considerable unmet clinical needs. It remains one of the most under-treated psychiatric disorders globally, with treatment coverage estimated at 10–20% [4]. A meta-analysis found that only up to 50% of individuals with AUD achieve remission over several years [5]. In response to the challenge of high relapse rates, innovative approaches such as mindfulness-based relapse prevention (MBRP) have emerged. MBRP integrates core aspects of relapse prevention with techniques adapted from mindfulness-based stress reduction (MBSR) and mindfulness-based cognitive therapy (MBCT) [6, 7]. At the core of MBRP lies the emphasis on identifying high-risk situations, training participants to recognize early warning signs for relapse, enhancing awareness of internal and external cues associated with substance use, developing effective coping skills, and boosting self-efficacy [8]. These strategies directly address the mechanisms underlying addiction, such as cue reactivity, loss of cognitive control, and impairment of interoceptive processing, as highlighted in numerous studies [9, 10].

The neuronal correlates of these mechanisms have been explored by means of neuroimaging such as functional magnetic resonance imaging (fMRI) and electroencephalography (EEG). Research has shown that MBRP and similar mindfulness-based interventions (MBIs) were found to increase resting state connectivity between the default mode network and prefrontal regions that contribute to cognitive control [11, 12]. In addition, after brief MBI, enhanced prefrontal activation during an executive attention task as well as increased glutamate metabolism in the anterior cingulate cortex (ACC) were observed [13, 14]. MBI also positively influenced behavioral inhibition, which correlated with enhanced conflict and response monitoring as indicated by neurophysiological EEG measurements [15, 16]. Additionally, MBIs have been effective in altering addiction-related attentional biases in individuals with alcohol use disorder [17].

In line with these findings, studies have observed decreased neuronal activation in response to drug-related cues in brain regions associated with cue reactivity such as the medial prefrontal cortex (PFC) and ventral striatum (VS) [18, 19]. Consistent with these results, altered functional connectivity in neurocircuitry related to craving such as between the insula and the VS has been found [20]. Functional neuroimaging of the effects of MBIs thus points towards a modulation of striatal cue reactivity, possibly through engagement of prefrontal control processes that result in reduced craving. Indeed, this lines up with results in an AUD cohort, showing that abstinence was positively associated with ACC-striatal connectivity during the presentation of alcohol cues [21].

Further underlining the importance of this brain region, ACC activation has also been linked to the processing of interoceptive cues [22]. The modification of interoception is hypothesized to be an important driver of the efficacy of MBIs [23]. Improved interoceptive processing could foster a more adaptive response to bodily sensations and enhance the capability to recognize and manage craving effectively [9, 24, 25].

Moreover, there is a strong link between frontal midline theta (FMΘ) oscillatory brain activity and MBI-induced increases of cognitive control. In patients with opioid use disorder, increased self-regulatory strength and self-control was indicated by endogenous theta stimulation in the PFC during active mindfulness meditation [26, 27]. FMΘ during behavioral inhibition has been linked to activity of a network that includes, among others, the ACC as well as medial PFC frontal gyrus [28].

Besides MBIs, neurotechnological interventions such as transcutaneous vagus nerve stimulation (tVNS), may improve cognitive control by modulating FMΘ oscillations. In analogy to the effects of mindfulness on neuronal function, it has been suggested that tVNS can modulate activity in prefrontal brain regions associated with cognitive control [29]. In line with this, FMΘ was found to be enhanced under tVNS along with behavioral improvements in executive control [30]. Furthermore, tVNS has been shown to improve measures of interoception [31, 32]. Considering this, tVNS has been suggested as a tool in the treatment of several psychiatric disorders and could be an effective adjunct to psychological modulation of self-regulation/cognitive control such as MBIs. Indeed, tVNS has been proposed to be an effective treatment approach in AUD [33]. Therefore, we reason that augmenting a MBRP program with tVNS could amplify improvements in cognitive function and interoceptive processing along with decreasing cue-associated behavior e.g. craving-associated drug-seeking in AUD.

Although MBI and tVNS have been used to improve cognitive control and inhibit addiction-related behavior by modulating FMΘ, they are not ideal for investigating the causal role of FMΘ in generating such behavior. To causally link FMΘ oscillations to behavior, they should be directly engaged by neuromodulation without relying on unspecific mechanisms [34]. While transcranial magnetic stimulation (TMS) targeting medial prefrontal brain regions can reduce cue-induced craving, TMS is not suitable for frequency-specific neuromodulation [35, 36]. A major challenge in establishing neuromodulation that adapts to ongoing, endogenous brain oscillations is the suppression of stimulation artifacts impeding continuous neurophysiological assessments [37]. By using advanced mathematical approaches [38–40] and stimulation protocols such as amplitude modulation [41], this challenge could be overcome allowing now for frequency- and phase-specific adaptive neuromodulation.

Here, we propose using such approach, specifically closed-loop transcranial alternating current stimulation (CLAM-tACS), to explore the causal role of FMΘ oscillations in addiction-related behavior. CLAM-tACS avoids sensory co-stimulation and allows for the parallel assessment and adaptation of brain oscillations at the target frequency during stimulation [42–45]. This system can enhance or suppress endogenous brain oscillations by adjusting the phase difference between CLAM-tACS and targeted oscillations [43]. Our planned study will use this approach to investigate the causal link between medial prefrontal brain activity and addiction-related behavior by modulating FMΘ oscillations.

In summary, we want to investigate the synergistic effects of MBRP and neuromodulation techniques in the context of AUD. We propose that the previously described addiction-related mechanisms could be targeted on a psychological and neurophysiological level through mindfulness training and neuromodulation. This combination could enhance the efficacy of MBRP and in turn improve the treatment of AUD. In addition, we will investigate the causal role of FMΘ via innovative real-time modification of target oscillations.

## Methods and analysis

### Study design

**Fig. 1:**
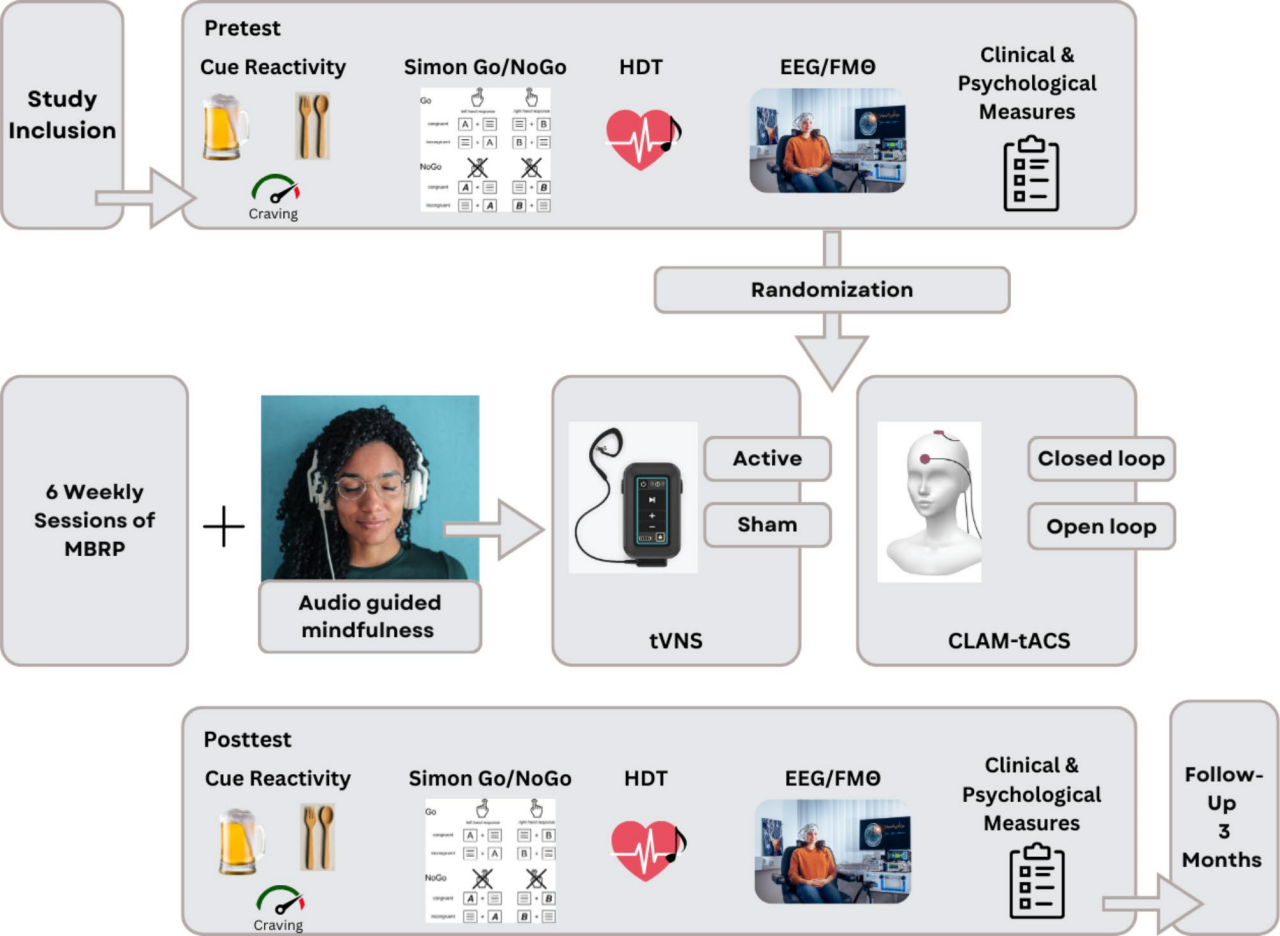
Graphical study design. Abbreviations: HDT – heartbeat detection task, EEG – electroencephalography, FMΘ – frontal midline theta, MBRP – mindfulness-based relapse prevention, tVNS – transcutaneous vagus nerve simulation, CLAM-tACS – closed-loop amplitude modulated transcranial alternating current stimulation. Image of Simon Go/NoGo task adapted from [46] licenced under CC BY 4.0

We will conduct a four-arm randomized controlled study at the Department of Psychiatry and Neurosciences, Charité – Universitätsmedizin Berlin. Detoxified patients with Alcohol Use Disorder (AUD) will undergo a meticulously structured Mindfulness-Based Relapse Prevention (MBRP) program consisting of six weekly group sessions supplemented with daily individual mindfulness practice sessions. Prior to and following treatment, participants will undergo comprehensive assessments including cognitive control evaluation using a Simon Go/NoGo (SGNG) task, assessment of cue-induced craving and a heartbeat discrimination task (HDT) as a marker for interoception, complemented by electrocardiographic measurements of heart rate and heart rate variability, alongside 32-channel electroencephalography (EEG) recordings. The pre and post treatment assessments will encompass various clinical and psychological metrics, including symptom and craving severity, as well as perceived loneliness and impulsivity. Long-term treatment effects will be evaluated through a follow-up assessment after three months, focusing on abstinence rates and alcohol consumption (Fig. 1).

### Recruitment

We will recruit patients with AUD in psychiatric clinics (inpatient and outpatient) throughout Berlin and Brandenburg. In addition, we will distribute our study information to support group networks that address patients with AUD. Interested patients will be screened for eligibility by means of a telephone interview.

### Inclusion criteria

- ICD-10 diagnosis of alcohol dependence (F10.2) and at least 2 criteria for AUD according to DSM-5 (last 12 months).
- Completion of in-patient withdrawal treatment and abstinence duration less than 12 months.
- Ability to understand study information and provision of written informed consent.
- Age 18–70 years.

### Exclusion criteria

- Epilepsy.
- Neurological disorders.
- Current episode of neuropsychiatric disorders such as schizophrenia spectrum disorders, major depressive disorders or bipolar affective disorders.
- Suicidality (acute).
- Use of antiepileptic or high-potent neuroleptic drugs.
- Current substance use disorder other than alcohol, nicotine and severe cannabis use disorder according to DSM-5.
- Implants (e.g., vagus nerve stimulator, cerebral shunt).

### Clinical measures

Several clinical as well as psychological measures will be included in the pre and post treatment assessments. Clinical interviews such as the SCID-5 as well as questionnaires will be used to evaluate patients’ drinking behaviour (retrospective quantity–frequency questionnaire as part of the M-CIDI (Munich Composite International Diagnostic Interview [47]), dependence severity (ADS [48]) as well as craving (AUQ [49], OCDS [50]). In addition, we will assess measures such as affective and anxiety symptoms (ADS [51], PANAS [52], STAI [53]), impulsivity (BIS-15 [54]), stress (TICS [55], PSS-10 [56]), trait and state mindfulness (FFA [57], FFMQ [58]). Furthermore, we will include various measures that could impact treatment outcome, such as abstinence self-efficacy (AASE [59]), psychological flexibility (PsyFlex [60]) and willingness to change (URICA [61]). All questionnaires will be completed before and after the intervention. To assess long-term treatment effects, patients will be followed-up after three months to assess abstinence days and alcohol consumption (heavy drinking days, total drinking days).

### MBRP

Detoxified AUD patients will be enrolled in a rolling format MBRP group therapy that consists of six weekly sessions and daily stand-alone practice of mindfulness techniques (independently performed by the participants between sessions). All participants will receive a psychological one-on-one consultation before start of MBRP to boost motivation to participate as well as convey general information about the structure of the program. MBRP group therapy will be administered by trained staff members and according to manuals by Bowen et al. [62] and Roos et al. [63] that were modified to accommodate six sessions in a rolling format. Further, group leaders receive regular/biweekly/monthly supervision by an experienced group psychologist/supervisor to ensure quality of MBRP group therapy during the study.

### Psychophysiological data

#### Tasks

Before and after treatment we will assess cognitive control with a hybrid of the Simon and go/no-go paradigms (Simon go/no-go; SGNG) that has been shown to engage frontal midline regions [46, 64]. Furthermore, we will assess physiological cue reactivity with a passive viewing task that adheres to the guidelines provided by Ekhitiari et al. [65], along with measures of subjective cue-induced craving. Finally, to evaluate the effect of tVNS on interoceptive accuracy, participants will complete a heartbeat discrimination task (HDT) that is based on the one that was developed by Critchley et al. [66].

#### EEG and HRV

32-channel EEG will be recorded using active Ag/AgCl electrodes (actiCap GmbH, Wörthsee, Germany). Recordings will be collected using a LiveAmp amplifier (Brain Products GmbH, Gilching, Germany). Data will be acquired at a sampling rate of 500 Hz and impedances will be kept below 10 kOhm. MNE-Python [67] will be used to analyze EEG data. The signal will be bandpass-filtered between 1 and 40 Hz, and the PREP pipeline [68] will be used to reject/repair bad sensors and segments. Morlet wavelets (1 to 40 Hz in 0.5 Hz steps, 7 cycles per wavelet) will be used to obtain a time-frequency transform of the data [69]. Finally, differences between conditions will be assessed using a cluster-based permutation test across all sensors and frequencies [70]. In this manner, we will assess whether changes in the EEG are specific to the theta frequency band.

### Neurostimulation sessions

#### tVNS

Once a week, each group will additionally receive a 30-minute tVNS/sham stimulation during an audio-guided body scan exercise. We will employ a tVNS-R device (tVNS International GmbH, Erlangen, Germany) to deliver transcutaneous electric stimulation to the inner side of the cymba concha for optimal targeting the auricular branch of the vagus nerve. For sham stimulation, the electrode will be placed on the ear lobe which contains no branches of the vagus nerve [71]. The stimulation frequency is set to 25 Hz, which is delivered in 30 s on/off phases. In increments of 100 μA the intensity will be increased individually until subjects report mild tingling.

#### CLAM- tACS

In analogy to the tVNS condition, CLAM-tACS will be conducted during a mindfulness exercise. We will employ a Digitimer DS5 (Digitimer Ltd, Hertfordshire, UK) to deliver amplitude-modulated transcranial alternating current stimulation (AM-tACS) through two circular rubber electrodes (4 cm ⌀) centered on positions Fpz and Cz of the international 10–20 system. Personalized current flow modeling will be used to optimize the tACS electrode montage and maximize target engagement (Kasten et al., 2019). The AM-tACS stimulation waveform will feature a carrier signal frequency of 8 kHz, an amplitude of ± 1 mA, and an envelope signal that is phase-tuned to frontal midline theta oscillations in real-time. Previous studies have shown that tACS at frequencies in the kHz range is a safe and effective approach to modulate cortical excitability [72]. EEG will be recorded using a 64-channel Bittium NeurOne system (Bittium, Oulu, Finland), which will deliver a real-time UDP stream to a Speedgoat Performance Real-Time target machine (Speedgoat GmbH, Liebefeld, Switzerland) that will apply artifact suppression and real-time phase estimation of frontal midline theta oscillations. The target machine will deliver the stimulation signal to the Digitimer DS5 in real-time. To assess phase-dependent effects of CLAM-tACS on FMΘ, we will bandpass-filter the data from 4 to 8 Hz using an FIR filter. Subsequently, SASS will be applied to suppress the stimulation artifact [43]. Finally, the Hilbert transform will be used to obtain instantaneous amplitude and phase of FMΘ. Instantaneous phase will be used to verify correct phase-locking between CLAM-tACS and FMΘ, and instantaneous amplitude will be used to assess enhancement or suppression of FMΘ.

### Data management and monitoring

The conduct of the investigation and processing of all personal data will be carried out in accordance with the latest version of the General Data Protection Regulation (GDPR). The pseudonymized research data essential for the study will be stored on servers at Center for Information Services and High Performance Computing (ZIH), Dresden. To collect data, we will employ REDCap, a certified data capture system that is compliant with Good Clinical Practice (GCP) standards and FDA 21 CFR Part 11. Access to the REDCap platform is protected by an authentication process. Only authorized study personnel with a personal identifier have access to the study server. Regarding hardware used for data collection purposes (PCs, laptops), only centrally supported systems are utilized. For study consent with identifying handwritten data, pseudonymized test sheets are archived separately in an access-secured database. The data storage duration is set for 10 years to facilitate the use of data for researching the long-term development of substance use and psychiatric disorders, which necessitates such a prolonged data storage period for follow-up surveys. All participants have the right to review the collected data as well as request the blocking or deletion of their data until the reidentification list is deleted.

### Sample size

For the examination of the effect of tVNS, we based our sample calculations on a study that investigated the effect of tVNS on protracted alcohol withdrawal symptoms in abstinent AUD patients [73]. According to the changes in the craving scores, considering a 95% confidence interval, 80% power (α = 0.05), and the related mean and standard deviation (SD) of the craving scores in the aforementioned study (μ1 = 4.82; μ2 = 3.92; SD1 = 0.49; SD2 = 0.39) we estimate *n* = 40 for each group. With an attrition rate of 20%, final estimated sample size is *n* = 50 per group. With regards to the application of CLAM-tACS, we performed a sample size estimation on the basis of a similar study investigating the causal role of frontal theta oscillations in a clinical population [74]. There, a medium effect size of dz = 0.577 was found, which corresponds to a sample size of *n* = 20 per group at a power of 0.8 und an alpha level of 0.05. In total we will recruit *n* = 140 AUD patients to be included in this study.

### Statistical methods and analysis

Our primary analyses will focus on the impact of active vs. sham stimulation (tVNS and CLAM-tACS) on neurobehavioral measures. To assess these effects, we will employ a linear mixed modelling approach to estimate the effects of treatment by time. Mediation will be assessed with path analyses.

First, we will investigate effects on cognitive function of augmented MBRP by administering the SGNG. Here, changes in SGNG behavioural inhibition pre to post treatment will be assessed, this further serves as a probe of the possible mediating effects of executive functioning on treatment success. We will further assess changes in cue reactivity as well as HDT interoceptive accuracy with multilevel modelling to allow for subject-specific effects [75]. Our study design also allows for the investigation of various levels of cue-induced effects, ranging from subjective indices of craving to physiological responses. Second, we will compare sham versus active stimulation + MBRP in terms of abstinence rates and drinking measures directly after and three months post treatment. We will investigate the course of clinical symptom severity as well as psychological measures before and after treatment as well as three months post treatment.

### Patient and public involvement

The feedback of a committee that consists of affected AUD patients and the Federal Association of the Families of Mentally ill People as well as speakers of addiction counseling centers in Berlin has been incorporated in the design of this study. In addition, we will collect feedback from all participating AUD patients at the end of their participation. This allowed us to adapt the design of the current study as closely as possible to the needs of those affected and to take experience gained from participating in our study into account for future projects.

## Discussion

This research project endeavors to enhance the effectiveness of MBRP while advancing treatment options for AUD. The study will delve into the nuanced impact and underlying mechanisms of neurostimulation protocols in conjunction with MBRP. Specifically, we will investigate the potential of tVNS and CLAM-tACS as supplementary treatments without incorporating a control intervention to MBRP. Numerous meta-analyses have highlighted the effectiveness of various mindfulness-based interventions (MBIs) in AUD populations, prompting our exploration into the mechanisms underlying this efficacy [76, 77]. While our study design does not allow for conclusive findings regarding potential disparities in cue reactivity, cognitive control, and interoceptive abilities between AUD individuals and healthy controls, we aim to monitor the potential influence of our intervention on these factors. Additionally, we anticipate that disease severity and treatment efficacy could be predicted based on these factors and their interplay, considering the inherent heterogeneity within AUD populations [78]. Conducting exploratory analyses on individual profiles could significantly contribute to a dimensional conceptualization, thereby facilitating the development of enhanced treatment and prevention strategies.

## Data Availability

No datasets were generated or analysed during the current study.

## Abbreviations

ACC: Anterior Cingulate Cortex
AASE: Abstinence Self–Efficacy Scale
ADS: Alcohol Dependence Scale
AUD: Alcohol Use Disorder
AUQ: Alcohol Urge Questionnaire
BIS-15: Barratt Impulsiveness Scale–15
CLAM-tACS: Closed–Loop Amplitude–Modulated Transcranial Alternating Current Stimulation
DSM-5: Diagnostic and Statistical Manual of Mental Disorders, 5th Edition
EEG: Electroencephalography
FMΘ: Frontal Midline Theta
FFA: Freiburg Mindfulness Inventory (FFI; German: Freiburger Fragebogen zur Achtsamkeit)
FFMQ: Five Facet Mindfulness Questionnaire
GCP: Good Clinical Practice
GDPR: General Data Protection Regulation
HDT: Heartbeat Detection Task
ICD-10: International Classification of Diseases, 10th Revision
MBRP: Mindfulness–Based Relapse Prevention
MBSR: Mindfulness–Based Stress Reduction
MBCT: Mindfulness–Based Cognitive Therapy
MBIs: Mindfulness–Based Interventions
M-CIDI: Munich Composite International Diagnostic Interview
OCDS: Obsessive Compulsive Drinking Scale
PANAS: Positive and Negative Affect Schedule
PFC: Prefrontal Cortex
PSS: 10–Perceived Stress Scale–10
PsyFlex: Psychological Flexibility Questionnaire
SCID-5: Structured Clinical Interview for DSM–5
SGNG: Simon Go/No–Go task
STAI: State–Trait Anxiety Inventory
TICS: Trier Inventory for Chronic Stress
TMS: Transcranial Magnetic Stimulation
tVNS: Transcutaneous Vagus Nerve Stimulation
URICA: University of Rhode Island Change Assessment
VS: Ventral Striatum
